# A Novel Hybrid Data-Driven Model for Daily Land Surface Temperature Forecasting Using Long Short-Term Memory Neural Network Based on Ensemble Empirical Mode Decomposition

**DOI:** 10.3390/ijerph15051032

**Published:** 2018-05-21

**Authors:** Xike Zhang, Qiuwen Zhang, Gui Zhang, Zhiping Nie, Zifan Gui, Huafei Que

**Affiliations:** 1 School of Hydropower and Information Engineering, Huazhong University of Science and Technology, Wuhan 430074, China; koko@hust.edu.cn; 2 School of Municipal and Mapping Engineering, Hunan City University, Yiyang 413000, China; zhipingnie@hotmail.com; 3 Key Laboratory for Digital Dongting Lake Basin of Hunan Province, Central South University of Forestry and Technology, Changsha 410004, China; quehuafei@hotmail.com; 4 Shenzhen Garden Management Center, Shenzhen 518000, China; guizifan@126.com

**Keywords:** daily land surface temperature, forecasting, data-driven, hybrid model, Ensemble Empirical Mode Decomposition (EEMD), Long Short-Term Memory (LSTM), Neural Network (NN), Dongting Lake basin

## Abstract

Daily land surface temperature (LST) forecasting is of great significance for application in climate-related, agricultural, eco-environmental, or industrial studies. Hybrid data-driven prediction models using Ensemble Empirical Mode Composition (EEMD) coupled with Machine Learning (ML) algorithms are useful for achieving these purposes because they can reduce the difficulty of modeling, require less history data, are easy to develop, and are less complex than physical models. In this article, a computationally simple, less data-intensive, fast and efficient novel hybrid data-driven model called the EEMD Long Short-Term Memory (LSTM) neural network, namely EEMD-LSTM, is proposed to reduce the difficulty of modeling and to improve prediction accuracy. The daily LST data series from the Mapoling and Zhijiang stations in the Dongting Lake basin, central south China, from 1 January 2014 to 31 December 2016 is used as a case study. The EEMD is firstly employed to decompose the original daily LST data series into many Intrinsic Mode Functions (IMFs) and a single residue item. Then, the Partial Autocorrelation Function (PACF) is used to obtain the number of input data sample points for LSTM models. Next, the LSTM models are constructed to predict the decompositions. All the predicted results of the decompositions are aggregated as the final daily LST. Finally, the prediction performance of the hybrid EEMD-LSTM model is assessed in terms of the Mean Square Error (MSE), Mean Absolute Error (MAE), Mean Absolute Percentage Error (MAPE), Root Mean Square Error (RMSE), Pearson Correlation Coefficient (CC) and Nash-Sutcliffe Coefficient of Efficiency (NSCE). To validate the hybrid data-driven model, the hybrid EEMD-LSTM model is compared with the Recurrent Neural Network (RNN), LSTM and Empirical Mode Decomposition (EMD) coupled with RNN, EMD-LSTM and EEMD-RNN models, and their comparison results demonstrate that the hybrid EEMD-LSTM model performs better than the other five models. The scatterplots of the predicted results of the six models versus the original daily LST data series show that the hybrid EEMD-LSTM model is superior to the other five models. It is concluded that the proposed hybrid EEMD-LSTM model in this study is a suitable tool for temperature forecasting.

## 1. Introduction

Accurate forecasting of daily land surface temperature (LST) is highly important for various fields, including weather maintenance services, agriculture, eco-environment, and industry [1]. Daily LST forecasting is the main forecasting factor in the daily weather forecast system [2]. In agriculture, daily LST forecasting can be adopted for agriculture irrigation systems, pest management schemes and diseases warring systems to predict the crop growth conditions that are useful for scheduling proper actions for drought development, as well as trends in the spread of diseases and pests [1,3,4]. Temperature (i.e., water temperature, soil temperature and Urban Heat Island, etc.) forecasting plays an important role in eco-environment-related research involving the functioning of the eco-environment system [5,6,7,8,9,10,11,12,13]. In industry, accurate forecasts of temperature are part of an energy-management strategy to reduce energy consumption while maintaining an internal temperature within a specified comfort range [14,15]. As the dramatic and continuous increase of rapid socio-economic development, population growth, and industrial, commercial and residential emissions of large amounts of heat have led to local temperature increases, they have in turn attracted attention from national governments and scientists [16,17,18,19,20]. However, daily LST variations are extremely nonstationary and nonlinear in nature, because the meteorological processes have been heavily impacted by global and local warming and climate change, as well as human activities [21]. Thus, improving prediction accuracy has been a major challenge. Therefore, the accurate daily LST forecasting model should be built to overcome these challenges.

Until now, many data-driven models have been proposed for time series forecasting. From different points of view, these models can be largely divided into four classes: climate models, statistical models, Neural Network (NN) models and hybrid models. Climate models are most widely used models for climate simulation, which apply quantitative methods to simulate the interactions of the important drivers of climate for various purposes, from study of the dynamics of the climate system to projections of future climate [18,22]. All of these climate models adopted the idea of energy balance. However, these models need many physical parameters gained from solar ongoing radiation energy, the earth’s outgoing surface radiation energy, the absorbed energy of surface cover and others from empirical data, etc. [18]. Due to the complexity of the physical processes of climate variations and the large amount of money and instruments required to obtain these data, the application of these models is limited, especially in developing countries. Statistical models, also known as Box-Jenkins models, are widely and successfully used for time series modeling and forecasting in recent decades, for example, the Auto-Regressive (AR) model, the Auto-Regressive Moving Average (ARMA) model and the Auto-Regressive Integrated Moving Average (ARIMA) [23]. However, these models require that the time series be stationary and have a large number of data points for a robust forecasting result. Nonlinear data-driven models, such as the Artificial Neural Network (ANN), with its advantage of learning and identifying complex data patterns with less data, has captured significant attention in precipitation, rainfall, runoff, drought, evapotranspiration and temperature forecasting problems in the past few years [24,25,26,27,28,29,30,31]. However, one of the major challenges faced by ANN is that it requires an iterative adjustment of model parameters, a slow response of the gradient-based learning algorithm used, and a relatively low prediction accuracy compared with more advanced NN algorithms [32,33,34]. Therefore, hybrid data-driven models, particularly in the last few years, have received much attention and have been widely adopted and applied in hydro-climate analysis to improve prediction accuracy as powerful alternative modeling tools. The hybrid models, especially the Wavelet Transform (WT) coupled with NNs, have provided promising levels of accuracy for time series forecasting, such as WT coupled with Back-Propagation Feed-Forward Multilayer perceptron (BP-FFML) [35], Artificial Neuron-Network Back-Propagation (ANN-BP) [36,37], Radial Basis Function (RBF) [38], Support Vector Machine (SVM) [39,40], Adaptive Neuro Fuzzy Inference System (ANFIS) [39], and so forth. However, WT requires and predetermines basis functions. Therefore, different basis functions can produce different results [41]. To solve this problem, a self-adaptive decomposition method has been introduced by Wu and Huang [42] for time series processing: The Ensemble Empirical Mode Decomposition (EEMD), which is based on the development of Empirical Mode Decomposition (EMD) [42,43]. Many hybrid methods that use a combination of EEMD and other algorithms have successfully been applied in some fields. For example, Wang et al. [44] utilized the EEMD coupled with the ARIMA for annual runoff time series forecasting. Zhang et al. [45] proposed a two-stage method that combined the EEMD with the multidimensional k-nearest neighbor model for financial time series forecasting. Niu et al. [46] applied the EEMD and the Least Square Support Vector Machine (LSSVM) base to Phase Space Reconstruction (PSR) for day-ahead PM_2.5_ concentration predictions. Wang et al. [30] proposed a hybrid model that utilized the EEMD coupled with ANN for long-term runoff forecasting. Zhang et al. [31] adopted the EEMD coupled with the Elman Neural Network (ENN) for annual runoff time series forecasting. Their research results demonstrated that the EEMD coupled with other popular methods can significantly improve time series forecasting precision compared with some other popular methods.

In this paper, a hybrid data-driven model, EEMD coupled with Long Short-Term Memory (LSTM), namely the EEMD-LSTM, is proposed for daily LST data series forecasting. Thus, the EEMD is employed to decompose daily LST data series into many relatively stable Intrinsic Mode Functions (IMFs) and one residue item. Then, the PACF algorithm is used to determine the number of inputs for LSTM models. Next, the decomposed results (IMFs and residue item) are modeled and forecasted using different LSTM models. The final predicted results are obtained by aggregating all the forecasted results of LSTM models. Finally, six statistical evaluation metrics (i.e., MSE, MAE, MAPE, RMSE, CC and NSCE) are used to measure the performance of the hybrid EEMD-LSTM compared with the hybrid EMD-RNN, EMD-LSTM and EEMD-RNN models and single RNN and LSTM models. In order to test this hybrid data-driven model, the daily LST data series from the Mapoling station in the Dongting Lake basin, central China, from January 1, 2014 to December 31, 2016 are used as a case study.

The reminder of this paper is organized as follows: Section 2 describes the EMD, EEMD, LSTM and the proposed hybrid EEMD-LSTM model in detail. Section 3 provides a case study in detail. Section 4 presents the conclusions of this paper.

## 2. Methodology Descriptions

### 2.1. Empirical Mode Decomposition (EMD)

Empirical Mode Decomposition (EMD) is a self-adaptive decomposition method which is developed for nonstationary and nonlinear signal processing [43]. Unlike Singular Spectrum Analysis (SSA), Fourier Transform (FT) and Wavelet Transform (WT), EMD does not require and predetermine basis functions and can decompose the original signal into many finite oscillation time scale components called IMFs and a residual component in a self-adaptive way [47]. Each IMF stands for the information on different scales of the original signal data series and must meet the following two rules: (1) In the whole signal data series, the number of extrema must be equal to the number of zero crossing or differ by one at most; (2) At any point, the mean value of the envelope defined by the local maxima and the minima must be zero.

Giving original signal data series x(t)(t=1, 2,…,n), the procedure of EMD can be described as follows:

1. Identify all the local maxima and minima of the original signal data series x(t).

2. Using the three-spline interpolation function to create the upper envelopes $e_{up}\left(t\right)$ and the lower envelopes $e_{low}\left(t\right)$ of the original signal data series.

3. Calculate mean value m(t) of the upper and lower envelopes. The mean value m(t) can be computed using the following formula:

(1) $$m\left(t\right)=\frac{e_{up}\left(t\right)-e_{low}\left(t\right)}{2},$$

4. Calculate the difference value d(t) between the original signal series x(t) and the mean value m(t). d(t) can be obtained through the following formula:

(2) d(t)=x(t)−m(t),

5. Check d(t): (a) if d(t) meets the two IMFs rules, then *d(t)* is defined as the ith IMF. The x(t) is replaced by the residue item r(t)=x(t)−d(t). Here, the ith IMF is represented as $c_{i}\left(t\right)$; (b) if d(t) does not meet the two rules, this means d(t) is not an IMF, so the x(t) is replaced by d(t).

6. Repeat steps 1 to 5, until the residue item r(t) becomes a monotone function or the number of extrema is less than one or equal to one, so that no more IMFs can be extracted. r(t) indicates the tendency of the original signal data series.

Finally, the original signal data series can be reconstructed through all the decomposition IMFs $c_{i}\left(t\right)$ and a residue r(t). It can be expressed as the following formula:

(3) $$x\left(t\right)=\sum_{i=0}^{n}c_{i}\left(t\right)-r\left(t\right),$$

The EMD method decomposes the original signal data series into many IMFs step-by-step from high frequency to low frequency and a trend item by self-adaptive, direct, complete, effective and approximately orthogonal, which doesn’t change the information and physical characteristics of the original signal data series. For original signal data series with data length N, it can be decomposed into log 2 N IMFs at most.

### 2.2. Ensemble EMD (EEMD)

Although the EMD method has many apparent advantages in processing nonstationary and nonlinear signal data, there also have some unavoidable defects [42]. The majority of these problems are: (1) endpoint effects and (2) mode-mixing. Endpoint-effects means that different ways of handling endpoint-effects in the EMD decomposition process will bring different results. Because the whole process is related to extrema points, it is very important whether the endpoint is an extrema value point. When the data are relatively short, the problem becomes even more pronounced. Mode-mixing refers to the fact that the same IMF contains different frequency components, or the frequency of the same and similar scale is distributed in different IMFs. So, the mode-mixing will not only cause the mixing of various scale vibration modes but can even lose the physical meaning of the individual IMF. In order to solve these problems of the EMD algorithm, a new Noise-Assisted Data Analysis (NADA) method is developed, namely Ensemble EMD (EEMD) [42]. The main procedure of EEMD method is expressed as follows:

1. Add white noise $w_{i}\left(t\right)$ to the original signal data series x(t). Then the new data series can be computed as follows:

(4) $$X_{i}\left(t\right)=x\left(t\right)-w_{i}\left(t\right),$$

2. Afterwards, decompose the new data series into IMFs using the EMD algorithm;

3. Repeat steps 1 and 2 with different white noises, adding to the original signal data series each time;

4. Obtain the mean of the ensemble corresponding IMFs of the decomposition results as the final results.

For the EEMD method, the first important step is to determine the ensemble times and the amplitude of adding noise. If the amplitude of added white noise is too small, it will probably not play a significant role in EMD decomposition. If it is too large, it will cause more interference and affect the results of the final decomposition. However, how to select the best ensemble times and the amplitude of adding noise is still an open question. Wu and Huang [42] suggest the amplitude of adding noise to 0.2 after comparing the results of the actual signal analysis. The effect of adding white noise should obey the following statistics rule:

(5) $$\varepsilon_{n}=\frac{\varepsilon }{\sqrt{N}},$$

where N is the number of ensemble times, ε represents the amplitude of the added noise and $\varepsilon_{n}$ is the final standard deviation of error, which is the difference between the original signal data series and the corresponding IMFs.

### 2.3. Long Short-Term Memory (LSTM) Neural Network

**Figure 1:**
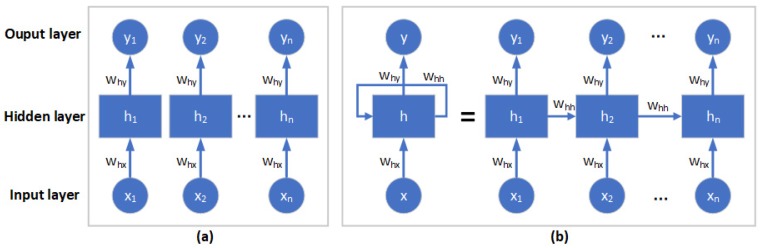
The architecture of (**a**) a traditional Artificial Neural Network (ANN) and (**b**) a Recurrent Neural Network (RNN).

The Recurrent Neural Networks (RNNs) are improved multilayer perceptron networks and somewhat different from those of traditional ANNs [48]. They have internal connections that can pass the processed signals at the current moment to the next moment. In RNNs model, each NN unit is connected with other hidden layers at different time steps, passing previous information to the current moment and computing with the input to form the output. Through loops in the hidden layer, information can thus be passed from one step to the next in the network (Figure 1). Because of the advantages of RNNs, the use of RNNs on many issues has achieved many incredible successes in the past few years, such as speech recognition, language modeling, translation, image captioning, and time series prediction [49,50,51].

**Figure 2:**
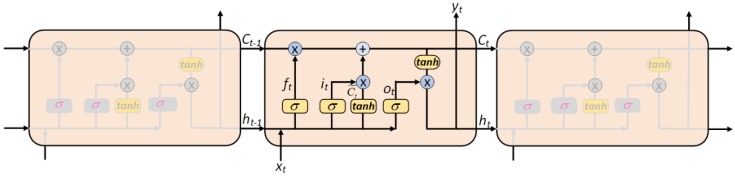
The architecture of the Long Short-Term Memory (LSTM) neural network.

Obviously, RNNs are suitable and able to process the complex long-term dependency problem in a simple way. However, RNNs tend to be severely affected by the vanishing gradient problem, which may increase indefinitely and eventually lead to network collapse [52]. Thus, simple RNNs may not be ideal for predicting long-term dependencies. To avoid this problem based on RNNs, Hochreiter and Schmidhuber [53] proposed a special type of RNN, namely the Long-Term Short Memory (LSTM) recurrent neural network. They were refined and popularized by many scholars. The architecture of LSMT is shown in Figure 2. As can be seen from Figure 2, the major advantage of LSTM is that LSTM replaces traditional neuron unit in the hidden layer of RNNs with a memory block, which has one or more memory cells and three adaptive multiplications known as the input gate, forget gate and output gate controlling the information flow through the cell and the neural network. Thus, the features and advantages of LSTM can effectively alleviate the vanishing gradient problem and makes it suitable for processing complex problems with long-term dependencies.

Figure 2 shows how the LSTM neural network works. The first step in LSTM is to determine whether information from the cell state is forgotten or remembered. This determination is made by a sigmoid layer called the forget gate layer. The output of forget gate is 0 (completely expunged) or 1 (completely retained). The calculating formula is as follows:

(6) $$f_{t}=\sigma \left(W_{f}\cdot \left[h_{t-1},x_{t}\right]+b_{f}\right),$$

The second step is to determine what new information needs to be stored in the cell state. This step consists of two parts. First, a sigmoid layer called the “input gate layer” determines which values are used for updating, and then, a tanh layer is used to generate a new candidate value $\tilde{C_{t}}$, which could be added to the cell state. At last, these two are combined to create an update to the state. The calculating formulas are expressed as follows:

(7) $$i_{t}=\sigma \left(W_{i}\cdot \left[h_{t-1},x_{t}\right]+b_{i}\right),$$

(8) $$\tilde{C_{t}}=tanh\left(W_{C}\cdot \left[h_{t-1},x_{t}\right]+b_{C}\right),$$

The third step is to update the old cell state $C_{t-1}$. First, we multiply the old cell state $C_{t-1}$ by $f_{t}$ to remove the information that we don’t need, we add $i_{t}*\tilde{C_{t}}$ to get the new candidate value, which scaled by how much we determine to update each state value. It can be calculated as the following formula:

(9) $$C_{t}=f_{t}*\;C_{t-1}+\;i_{t}*\tilde{C_{t}},$$

The final step is to determine the output of the model. First, we run a sigmoid layer to determinate what parts of the cell state we’re going to output, and then we put the cell state through tanh function and multiply it by the output of the sigmoid gate. The calculating formulas are defined as follows:

(10) $$o_{t}=\sigma \left(W_{o}\cdot \left[h_{t-1},x_{t}\right]+b_{o}\right),$$

(11) $$h_{t}=o_{t}*\tanh\left(C_{t}\right),$$

where in Equations (6)–(11), $x_{t}$ is the input at time t; $h_{t-1}$ and $h_{t}$ *t* are the outputs of the hidden layer at time t−1 and t, respectively; $C_{t}$ and $C_{t-1}$ are the cell output states at time t−1 and t, respectively; $\tilde{C_{t}}$ is the cell input state at time t. $f_{t}$, $i_{t}$ and $o_{t}$ are the outputs of the forget gate, input gate and output gate at time t, respectively; $W_{f}$, $W_{i}$, $W_{o}$ and $W_{C}$ are the weights connecting $h_{t-1}$ and $x_{t}$ to the forget gate, input gate, output gate and the cell input, respectively; $b_{f}$, $b_{i}$,$\;b_{o}$ and $b_{C}$ are their corresponding bias terms. σ denotes the sigmoid function $\frac{1}{1+\exp\left(-x\right)}$ and tanh indicates the hyperbolic tangent function $\frac{\exp\left(x\right)-\exp\left(-x\right)}{\exp\left(x\right)+\exp\left(-x\right)}$.

### 2.4. The Novel Hybrid EEMD-LSTM Data-Driven Model

**Figure 3:**
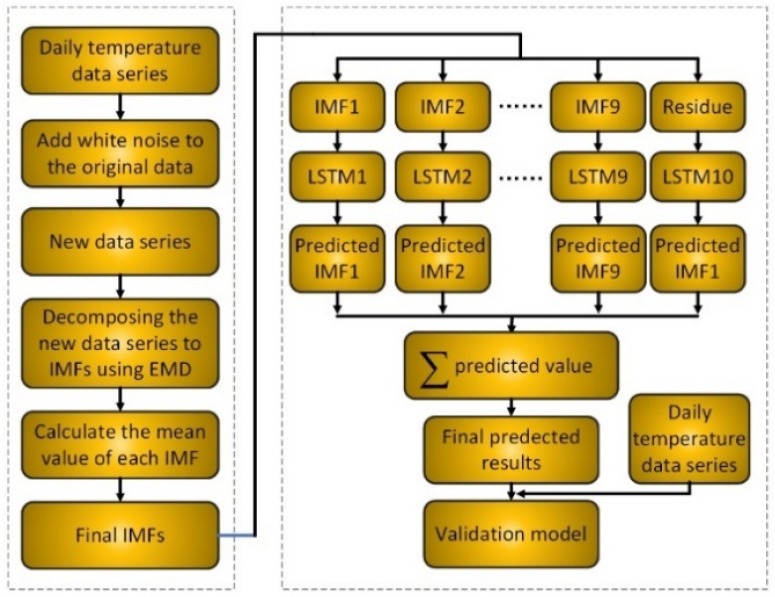
The architecture of the proposed Ensemble Empirical Mode Decomposition (EEMD)-LSTM neural network hybrid data-driven model.

Meteorological data series often shows different frequencies that can be nonstationary and nonlinear. Therefore, it is difficult to accurately model and forecast using a simple model. Thus, a hybrid model based on EEMD method and LSTM neural networks, namely EEMD-LSTM, is proposed to improve the prediction accuracy to solve and improve the long-term dependencies forecasting problem of daily LST. The EEMD method is firstly used to decompose the daily LST data series into many relatively stable IMFs and a residue item to reduce the difficulty of modeling. Then, all decomposed results are forecasted using the LSTM neural network. Finally, all of the forecasting results of decompositions are accumulated as the final predicted results. The workflow chart of the proposed hybrid EEMD-LSTM model is clearly shown in Figure 3. The main procedures of the EEMD-LSTM are as follows.

1. Daily LST data series decomposing. The original daily LST data series is decomposed into many IMFs and a residue item using the EEMD method.

2. Number of inputs determining. The PACF algorithm is used to gain the number of inputs of all the LSTM models.

3. IMFs and residue item modeling and forecasting. All the decomposition results are divided into two parts: the training data set and testing data set. The training data set is used for LSTM modeling. The testing data set is input into the trained LSTM models to predict all the IMFs and residue item. Then, many predicted IMFs and residue item results are achieved.

4. Final predicted results reconstructing. All the predicted results are accumulated as the final predicted results of the daily LST.

5. Model performance evaluation. Several statistical evaluation metrics are applied to assess the hybrid data-driven model between the predicted results and the original daily LST data series.

## 3. Case Study

### 3.1. Study Area

**Figure 4:**
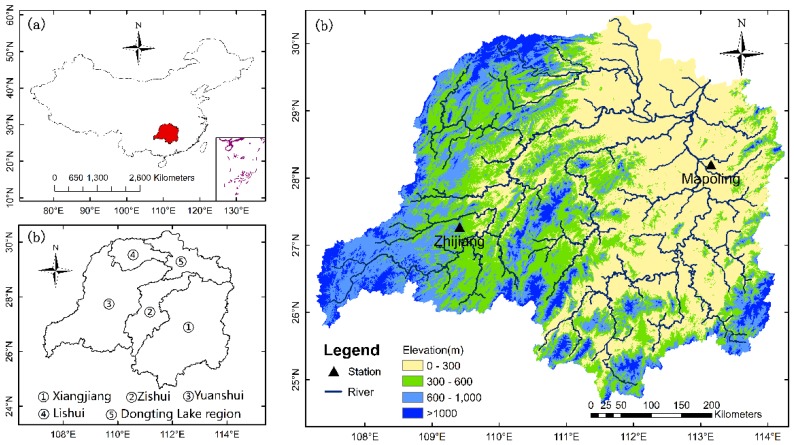
(**a**) Location of the Dongting Lake basin in central south China; (**b**) Composition of the basin; (**c**) Distribution of the Mapoling and Zhijiang meteorological stations.

The Dongting Lake basin is situated in the middle and lower reaches of the Yangtze River basin in the central south of China and lies approximately between the longitude of 107°16’ E~114°15’ E and the latitude of 24°38’ N~30°24’ N (Figure 4) [54]. It can be clearly seen from the Figure 4b that the Dongting Lake basin consists of four main rivers, including the Xiangjiang river, Zishui river, Yuanshui river and Lishui river, which flows through the six provinces of Guangdong, Guangxi, Guizhou, Jiangxi, Hubei and Hunan, discharging water into the Yangtze River through the Chenglingji outlet [55]. The Dongting Lake basin has a total drainage area of 26.3 × 10 4 km^2^, accounting for 14.6% of the total drainage area of the Yangtze River basin [31]. It can be clearly seen from Figure 4c that the topography of the basin is dominated by mountains and hills and varies from mountainous and hilly areas in the south, west, southwest and east to the alluvial plains in the central, north and northeast. The basin is in a subtropical monsoon climate zone with high temperatures and high levels of rainfall in summer, as well as low temperatures and less rain in winter. The annual precipitation level is from approximately 1300 mm to 1800 mm and the annual average temperature ranges from 16 °C to 18 °C [31].

### 3.2. Data Collection

**Figure 5:**
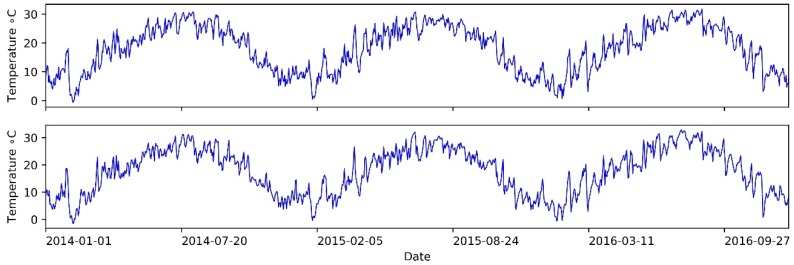
Daily land surface temperature (LST) data series of Mapoling station (**upper**) and Zhijiang station (**lower**) from 1 January 2014 to 31 December 2016.

In this study, daily LST data from the Mapoling and Zhijiang stations were obtained from the China Meteorological Data Sharing Service System (http://data.cma.cn) during 1 January 2014 to 31 December 2016. All the daily LST data are the daily average data of the four measuring times (2:00, 8:00, 14:00, 20:00), which have undergone a series quality control by the China Meteorological Administration (CMA), including the extreme values’ check and the internal consistency check. The accuracy rate of the daily LST data was generally more than 99%. The obtained daily LST data series are used to construct the hybrid EEMD-LSTM model and evaluate the model’s performance. The Mapoling station is located on the lower reaches of Xiangjiang river, in Changsha city, near the Dongting Lake, while the Zhijiang station is located on the mountain areas upper reaches of Yuanshui river, in Zhijiang county. We collected 1096 daily LST observation sample points, which are included in this study. The daily LST data series is shown in Figure 5. It is clear from the figure that the daily LST data series shows fluctuation characteristics. The whole data set is separated into the training data set and the testing data set. The training data set covering 1 January 2004 to 30 June 2016 is used for constructing models, while the testing data set ranges from 1 July to 31 December 2016 is used for assessing the prediction performance of the models.

### 3.3. Statistical Evaluation Metrics for Forecasting Performance

Six commonly and highly statistical evaluation metrics are employed to assess the prediction performance of the hybrid EEMD-LSTM model in this study. They are the mean squared error (MSE), Mean Absolute Error (MAE), Mean Absolute Percentage Error (MAPE), Root Mean Square Error (RMSE), Pearson Correlation Coefficient (CC) and Nash-Sutcliffe Coefficient of Efficiency (NSCE). The MSE is commonly used for measuring the degree of difference predicted and original data (Equation (12)). The MAE is a measure of the difference between predicted and original data (Equation (13)). The MAPE is selected for assessing the percentage deviation between predicted and original data (Equation (14)). The RMSE, as one of the most widely, frequently and commonly applied metrics, is used to measure the difference between values predicted by model and the actually observed (Equation (15)). The smaller the RMSE value is, the closer the predicted data are to the original data. The CC is a frequently and widely used indicator for measuring how well the predicted data correspond to the original data (Equation (16)). A CC equal to 0 indicates no or weak linear correlation, while a CC is closer to −1 or 1 indicates negative or positive linear correlation, respectively. The NSCE, proposed by Nash and Sutcliffe (1970), is one of the most powerful and popular evaluation indicators for assessing the power of hydro-climate models (Equation (17)). The NSCE value ranges from negative infinity and 0. An NSCE value of 1 corresponds to a perfect match of the model’s predictions to the original data. An NSCE of 0 indicates the model predictions are as accurate as the mean of the original data, whereas an NSCE less than 0 indicates the model is not trustworthy. Essentially, the closer the model NSCE is to 1, the more accurate the model is.

(12) $$MSE=\frac{1}{n}\sum_{i=1}^{n}{\left(T_{i}^{o}-T_{i}^{p}\right)}^{2},$$

(13) $$MAE=\frac{1}{n}\sum_{i=i}^{n}\left|T_{i}^{o}-T_{i}^{p}\right|,$$

(14) $$MAPE=\frac{1}{n}\sum_{i=i}^{n}\left|\frac{T_{i}^{o}-T_{i}^{p}}{T_{i}^{o}}\right|\times 100,$$

(15) $$RSME=\sqrt{\frac{1}{n}\sum_{i=1}^{n}{\left(T_{i}^{o}-T_{i}^{p}\right)}^{2}},$$

(16) $$CC=\frac{\sum_{i=1}^{n}(T_{i}^{o}-\bar{T^{o}})\left(T_{i}^{p}-\bar{T^{p}}\right)}{\sqrt{\sum_{i=1}^{n}{\left((T_{i}^{o}-\bar{T^{o}}\right)}^{2}}\sqrt{\sum_{i=1}^{n}{\left(T_{i}^{p}-\bar{T^{p}}\right)}^{2}}},$$

(17) $$NSCE=1-\frac{\sum_{i=1}^{n}{\left(T_{i}^{o}-T_{i}^{p}\right)}^{2}}{\sum_{i=1}^{n}{\left((T_{i}^{o}-\bar{T^{o}}\right)}^{2}},$$

where in Equations (12)–(17), $T_{i}^{o}\;$and $T_{i}^{p}$ are the original and predicted daily LST data series at time i, respectively. Whereas $T^{o}$ and $T^{p}$ are the mean value of original and predicted daily LST data series at time i, respectively. *n* represents the number of data sample points.

### 3.4. Daily LST Data Series Decomposition by EEMD

**Figure 6:**
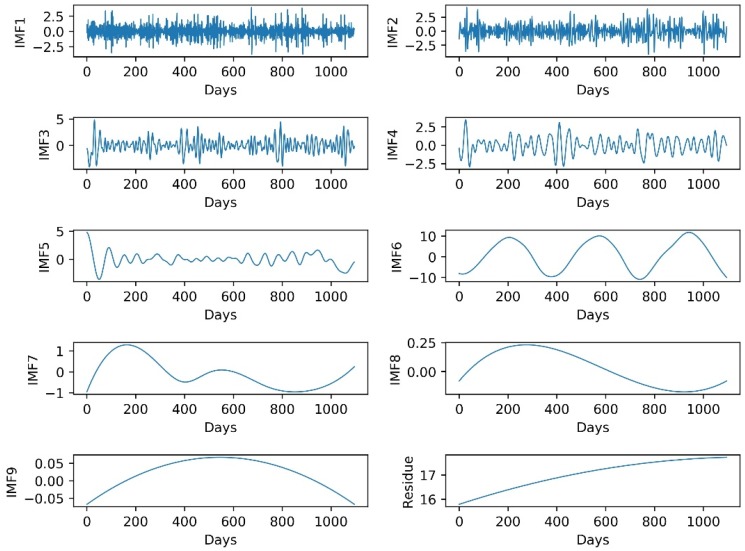
Decomposition results of the original daily LST data series of Mapoling station by EEMD.

**Figure A1:**
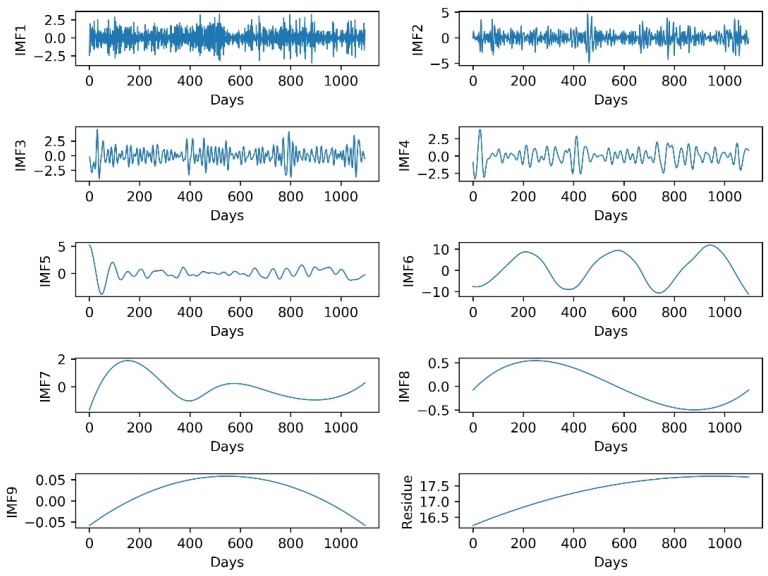
Decomposition results of the original daily surface temperature data series of Zhijiang station by EEMD.

EEMD is an excellent and powerful method for conducting nonstationary and nonlinear signal analysis. It decomposes the original data series into many relatively stable IMFs and one residue item. In the current study, the ensemble number is set to 1000 and the amplitude of added noise is set to 0.2 times the standard deviation of the corresponding data to decompose the daily LST data series of the two stations. Nine independent IMFs and one residue item from each station are obtained (Figure 6 and Figure A1). As can be seen from Figure 6 and Figure A1, IMF presents the oscillation characteristics in the order from high frequency to low frequency at various time scales and the last item is the overall trend of the original daily LST data series. Table 1 and Table A1 give the statistics of the original daily LST data series and decomposition results of the two stations. It is evident that the variance and standard deviation of the original daily LST data series from Mapoling and Zhijiang stations are 67.8232 and 8.2355, and 63.8016 and 7.9876, respectively. In contrast, the variance and standard deviation of all the decomposition results (every IMF and one residue item) are much smaller than the original daily LST data series. This indicates that the decomposition results have less volatility and are closer to their mean values. The skew of the original daily LST data series and decomposition results are closer to zero, indicating that the distribution of the data is approximately symmetric. While most kurtosis of the original daily LST data series and decomposition results are much smaller, indicating that the data have less extreme values. Therefore, the EEMD can be a powerful method to decompose nonstationary and nonlinear daily LST data series into many relatively stable IMFs for improving the prediction accuracy.

### 3.5. Forecasting IMFs

**Figure 7:**
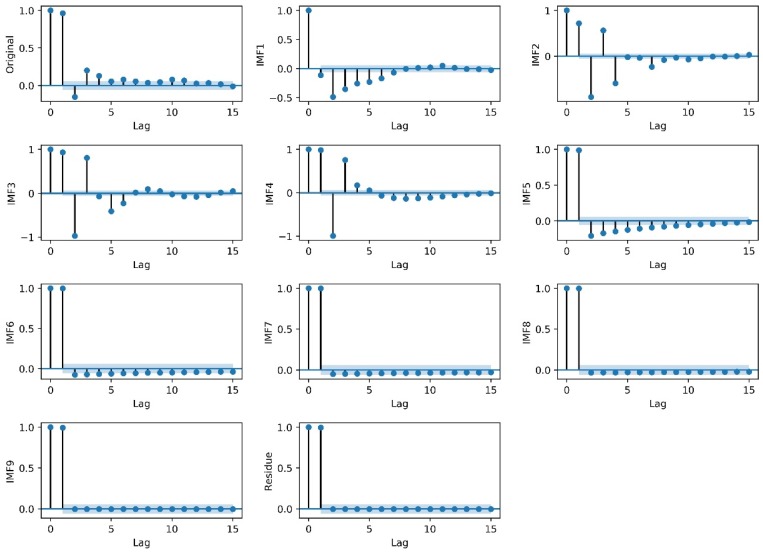
PACF graphs for the original daily LST data series and the decomposition results of the Mapoling station.

**Figure A2:**
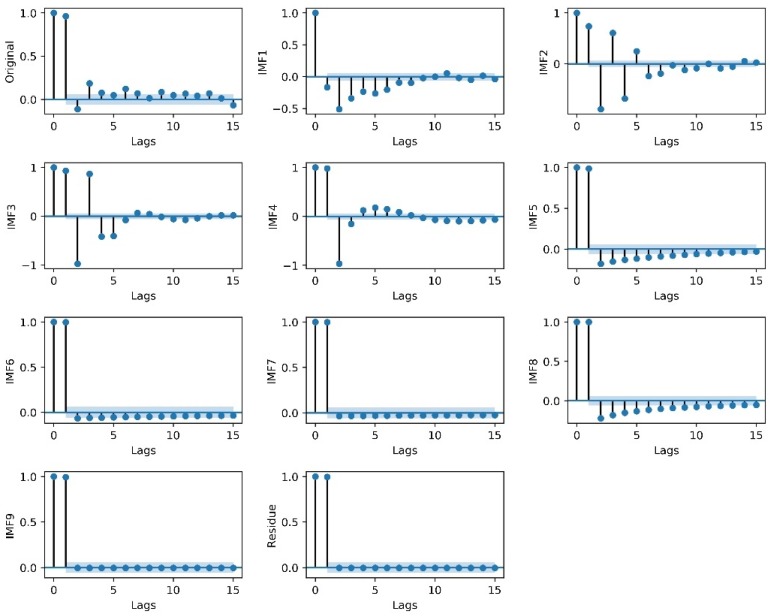
PACF graphs for the original daily surface temperature data series and the decomposition results of the Zhijiang station.

To improve the prediction accuracy, a four-tier layer LSTM is built to predict the daily LST data series and the decompositions (IMF1 to IMF9 and one residue item) in this study. However, the question of how to determine the appropriate number of inputs is still a key issue. In general, a common method of identifying the number of inputs is empirical. In this study, the Partial Autocorrelation Function (PACF) is used to analyze the original data and the decomposition results [56]. This is because the PACF can effectively identify the correlation between the current value and the previous values. A PACF value beyond the 95% confidence level indicates a strong correlation degree; otherwise there is a weak correlation degree. Therefore, the number of lags beyond the 95% confidence level is considered as the number of inputs. The PACF graphs of the original daily LST data series and their decomposition results of Mapoling and Zhijiang stations is shown in Figure 7 and Figure A2, respectively. Evidently the number of inputs of LSTM models for the original daily LST data series and their decomposition results of the Mapoling station are shown as 4, 6, 5, 5, 6, 6, 1, 1, 1, 1 and 1, respectively. While 4, 7, 8, 5, 6, 7, 1, 1, 7, 1, 1 are shown for Zhijiang station. Obviously, the number of inputs of each LSTM model is different. Since, the first several IMFs have high frequencies, the current value is related to many previous values. As the frequency decreases, the IMFs become more and more stable, and the current value is related only to its former one.

After the determination of the number of inputs of LSTM models, one-step-ahead is used to predict the results. That is, several previous data sample points are used to predict the current data point. The LSTM model consists of one input layer with several inputs which is determined by PACF before, for example, up to several previous ($x_{t-1},x_{t-2},\ldots ,x_{t-n}$) sample points of the original daily LST data series and the decomposition results are set as the model inputs; two hidden layers including 32 neurons each; and one output layer having one output, for example, $x_{t}$ is the current value of predicted results. Next, the LSTM model is implemented with TensorFlow which is an opensource and widely used neural network framework developed by Google [57]. In addition, the epoch for training is set to 4000 and in each training period, the MSE is employed as the loss function for determining the optimum performance results. Furthermore, the predicted results of the decomposition IMFs are obtained. Finally, all the predicted results are aggregated as the final prediction results of the daily LST data.

### 3.6. Performance Comparison Analysis

**Figure 8:**
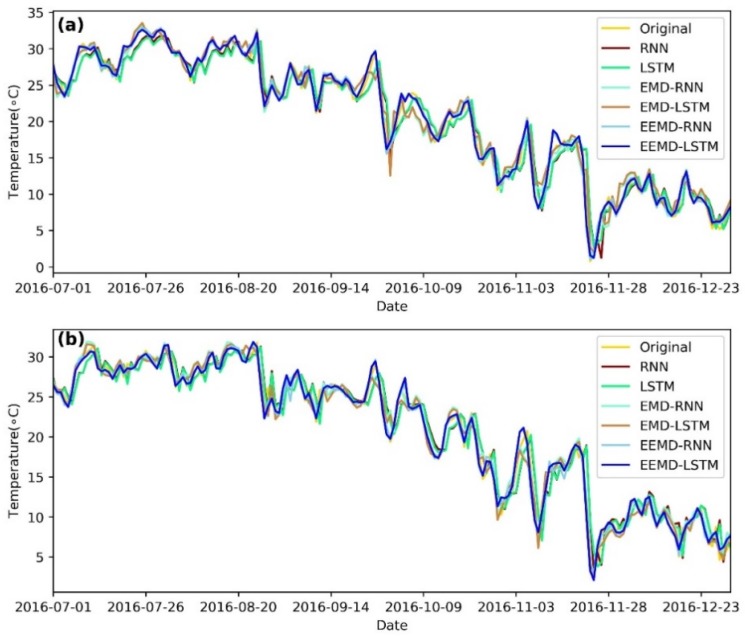
Performance comparison of the forecasting results of (**a**) Mapoling station and (**b**) Zhijiang station among RNN, LSTM, Empirical Mode Decomposition (EMD)-RNN, EMD-LSTM, EEMD-RNN and EEMD-LSTM.

To understand the performance of the hybrid EEMD-LSTM model, the predicted results of the hybrid EEMD-LSTM model are compared with the RNN, LSTM, EMD-RNN, EMD-LSTM and EEMD-RNN five models. The predicted results of the six models are illustrated in Figure 8. Obviously, the six models give different forecast results of the daily LST data series of the Mapoling and Zhijiang stations. But the hybrid EMD-RNN, EMD-LSTM, EEMD-RNN and EEMD-LSTM models perform better than single RNN and LSTM models for the two stations. Furthermore, the hybrid EEMD-LSTM model has a more powerful forecasting capacity, particularly when there have sudden changes in the data series. The reason is that the original daily LST data series are characteristic with nonstationary and nonlinear. There have been lots of sudden changes in the original data series. Thus, single RNN and LSTM models can hardly catch the sudden changes in the original data series. While the EMD decomposition results exit the drawbacks of edge-effects and mode-mixing. However, EEMD has overcome these drawbacks. Therefore, the hybrid EEMD-LSTM model achieves the highest accuracy for one-step-ahead forecasting compared with the other models.

**Figure 9:**
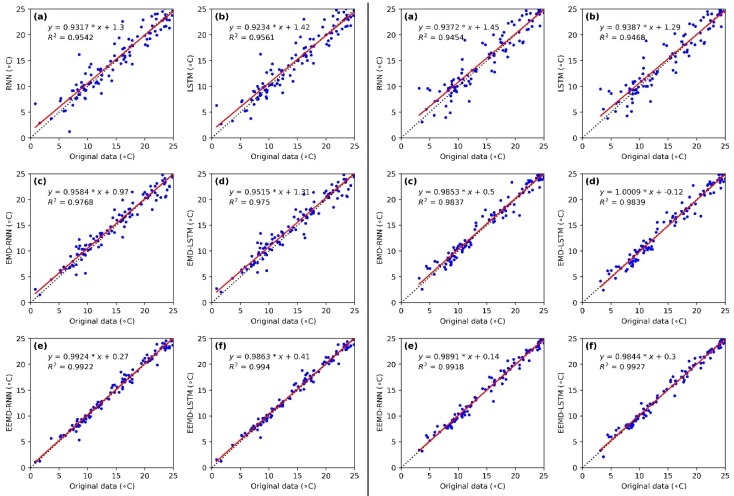
Scatterplot of the daily LST comparison of Mapoling station (**left**) and Zhijiang station (**right**) between (**a**) original data and RNN; (**b**) original data and LSTM; (**c**) original data and EMD-RNN; (**d**) original data and EMD-LSTM; (**e**) original data and EEMD-RNN; (**f**) original data and EEMD-LSTM from 1 July to 31 December 2016.

The scatterplots of the predicted results of the RNN, LSTM, EMD-RNN, EMD-LSTM, EEMD-RNN and EEMD-LSTM models versus the original daily LST data series in Mapoling station and Zhijiang station from 1 July 2016 to 31 October 2016 are shown in Figure 9. In general, it is obvious that the fitted lines (red line) of the predicted results of the six models are close to the 1:1 line (dot black line), which indicates that all the six models present high performance accuracy. Evidently, the RNN has the worst prediction results for the daily LST, while the LSTM obtains slightly better results than the RNN. However, the hybrid models (i.e., EMD-RNN, EMD-LSTM, EEMD-RNN and EEMD-LSTM models) perform better compared with the single RNN and LSTM models. The EEMD-LSTM model outperforms the other hybrid models with the highest coefficient of determination (R^2^) value for the two sites.

**Figure 10:**
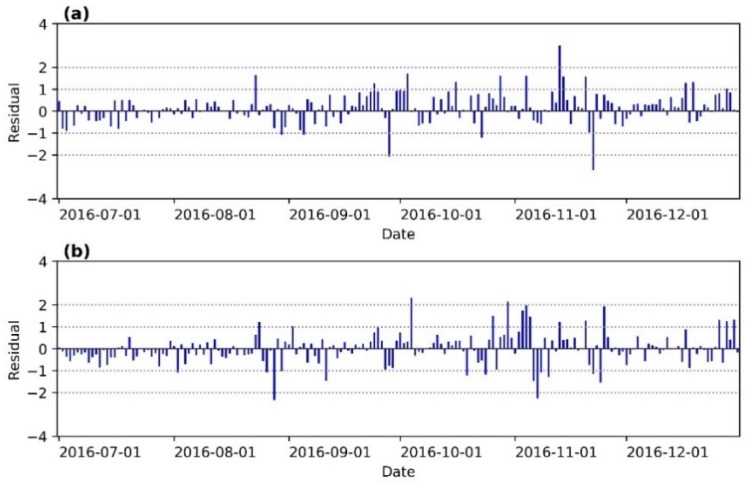
Bar plots of the residuals of the (**a**) Mapoling station and (**b**) Zhijiang station for original vs. EEMD-LSTM.

**Figure A3:**
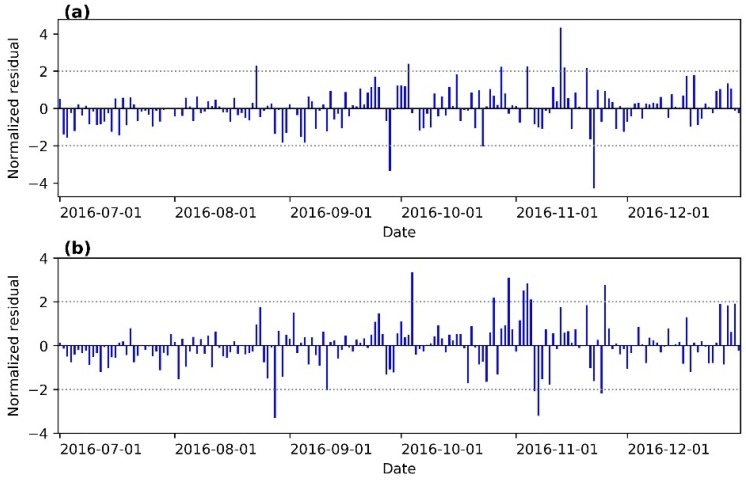
Bar plots of the residuals of the (**a**) Mapoling station and (**b**) Zhijiang station for original vs. EEMD-LSTM. The dotted lies are the confidence level at 95%.

To demonstrate the prediction capability of the EEMD-LSTM model, residual analysis is applied in this study. We calculate the residuals and normalized residuals of the two stations for original data vs. EEMD-LSTM (Figure 10 and Figure A3). Evidently, most of the residuals are between −1 and 1, and most of the normalized residuals are between the confidence level of 95%. But there are a few residuals and normalized residuals beyond the −1 and 1, and 95% confidence level. The potential reason for this is sudden changes in the original daily LST data series. Moreover, the prediction results close to the training data set have less residuals and normalized residuals, while far from the training data set have large residuals and normalized residuals. In order to obtain high prediction results, therefore, we suggest that the time span of daily LST data series prediction should not exceed three months. Otherwise, it is recommended to retrain the EEMD-LSTM model. Furthermore, compared with Figure 8, the daily LST data series are more stationary, the residuals are smaller and the prediction results are more perfect and trustworthy.

**Figure 11:**
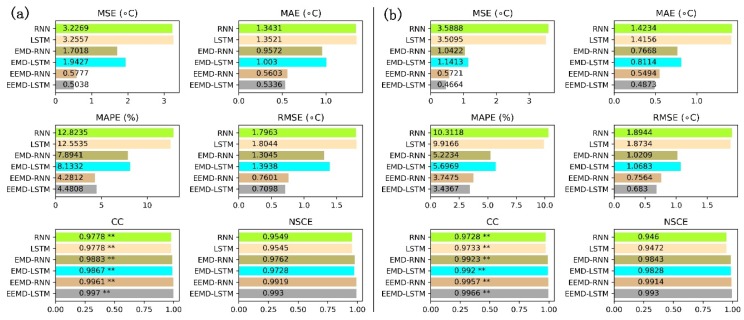
Bar charts of the statistical summary of the six models’ prediction results versus the original daily LST data series of (**a**) Mapoling stationand (**b**) Zhijiang station (** indicating a significance level of 0.01).

To further assess the prediction performance of the hybrid EEMD-LSTM model, six statistical evaluation metrics (i.e., MSE, MAE, MAPE, RMSE, CC and NSCE) are utilized to measure performance. The statistical evaluation results of performance comparison of the six models for daily LST data series are shown in Figure 11. According to the comparison of the RNN, LSTM, EMD-RNN, EMD-LSTM, EEMD-RNN and EEMD-LSTM models for the Mapoling and Zhijiang stations, all the six models clearly show high performance accuracy with the CC values greater than 0.97. Meanwhile, the CC values of the six models are significant at the significance level of 0.01. This means that the prediction results of the six models significantly correlate with the original daily LST data series and have the potential to predict the daily LST. Among all the six models, it is evident that the RNN model has the worst performance results compared with the other models. The LSTM model performs slightly better than the RNN model. The reason for the poor performance of the RNN and LSTM modes is the nonstationary and nonlinear nature of the original daily LST data series. However, the hybrid EEMD-LSTM model outperforms the other models with the smallest MSE, MAE, MAPE and RMSE, as well as the largest CC and NSCE for daily LST forecasting. Furthermore, the NSCE values of the six models are close to 1. This indicates that the predicted results of the six models perfectly match the original daily LST data series and the six models are trustworthy. However, the hybrid EEMD-LSTM has the largest NSCE value, which indicates that the hybrid EEMD-LSTM model is superior to the RNN, LSTM, EMD-RNN and EMD-LSTM models and is the best suitable model for daily LST forecasting.

Depending on the comparison of the aforementioned six models, we can reach the conclusion that using the EEMD method to decompose the original daily LST data series to many relatively stable IMFs and one residue item as the input for LSTM models can, to a large extent, improve the prediction accuracy. Thus, the proposed EEMD-LSTM model is a better model than the RNN, LSTM, EMD-RNN, EMD-LSTM and EEMD-RNN models and can achieve better predicting results with a significant improvement on the basis of six statistical evaluation metrics for daily LST forecasting.

## 4. Conclusions

In this study, we proposed a hybrid data-driven model based on EEMD and four-layer LSTM models to predict the daily LST data series. The daily LST data series from the Mapoling station located on the lower reaches of the Xiangjaing river and Zhijiang station located on the upper reaches of Yuanjiang river in Dongting Lake basin, central south China, from 1 January 2014 to 31 December 2016 are used as a case study. The main conclusions of this study are as follows: (1) the original daily LST data series are decomposed into nine relatively stable IMFs and one residue item using the EEMD method to reduce the difficulty of modeling and improving the prediction accuracy. Then, all the decomposition results are divided into the training data set and the testing data set. Next, the PACF algorithm is employed to choose the best number of inputs. After the best number of inputs is determined, the training data set is used to construct the LSTM models and the testing data set is used for predictions and performance comparisons. Finally, the predicted results of the decompositions are obtained and aggregated as the final prediction of the daily LST data. (2) Six statistical evaluation metrics (MSE, MAE, MAPE, RSME, CC and NSCE) are adopted to assess the performance of the RNN, LSTM, EMD-RNN, EMD-LSTM, EEMD-RNN and EEMD-LSTM models. The performance comparison of prediction results in this study shows that all the six models have high prediction accuracy. But the hybrid EEMD-LSTM model has performs better than the RNN, LSTM, EMD-RNN, EMD-LSTM and EEMD-RNN models. While, the hybrid EEMD-LSTM obtained a perfect prediction results for daily LST data series forecasting, the model needs additional future studies in other regions in mainland China. In brief, developing a hybrid data-driven forecasting model by using the LSTM coupled with EEMD algorithm may significantly improve the prediction accuracy.

## Figures and Tables

**Table 1:** Statistics of the original daily LST data series and the decomposition results of Mapoling station.

Series	Period	Min.	Max.	Mean	Variance	SD ^1^	Skewness	Kurtosis
Original data set	1 January 2014 to 31 December 2016	−1.5	32.8	17.6599	67.8232	8.2355	−0.2036	−1.0706
	1 January 2014 to 30 June 2016 (Training)	−1.5	32	17.0957	65.1861	8.0738	−0.2059	−1.0738
	1 July 2016 to 31 December 2016 (Testing)	0.8	32.8	20.4565	71.4957	8.4555	−0.3571	−1.1502
IMF1	1 January 2014 to 31 December 2016	−3.7604	3.9356	−0.0045	1.076	1.0373	0.0456	1.2377
	1 January 2014 to 30 June 2016 (Training)	−3.7604	3.9356	-0.0047	1.1645	1.0791	0.0466	0.9976
	1 July 2016 to 31 December 2016 (Testing)	−2.9097	2.8863	−0.0037	0.6374	0.7984	0.0279	2.92
IMF2	1 January 2014 to 31 December 2016	−4.1524	4.2432	−0.008	1.508	1.228	0.0174	0.5498
	1 January 2014 to 30 June 2016 (Training)	−4.1524	4.2432	−0.0063	1.4944	1.2224	0.0309	0.4341
	1 July 2016 to 31 December 2016 (Testing)	−4.1085	3.6196	−0.0162	1.5756	1.2552	−0.0441	1.1147
IMF3	1 January 2014 to 31 December 2016	−4.1166	4.8691	−0.0506	1.734	1.3168	0.0441	1.1287
	1 January 2014 to 30 June 2016 (Training)	−4.1166	4.8691	-0.0763	1.6987	1.3034	0.0537	1.3837
	1 July 2016 to 31 December 2016 (Testing)	−3.9231	3.6299	0.0768	1.8891	1.3745	-0.025	0.1554
IMF4	1 January 2014 to 31 December 2016	−2.9359	3.4556	−0.0027	1.1967	1.0939	−0.0078	0.0501
	1 January 2014 to 30 June 2016 (Training)	−2.9359	3.4556	-0.0072	1.2543	1.12	0.0216	0.0981
	1 July 2016 to 31 December 2016 (Testing)	−2.1632	2.0125	0.0197	0.9102	0.9541	−0.2184	−0.7181
IMF5	1 January 2014 to 31 December 2016	−3.5915	4.826	−0.044	1.2316	1.1098	0.0722	3.0066
	1 January 2014 to 30 June 2016 (Training)	−3.5915	4.826	0.0478	1.0681	1.0335	0.294	5.0861
	1 July 2016 to 31 December 2016 (Testing)	−2.4797	1.6551	−0.499	1.7933	1.3392	0.0258	−1.2748
IMF6	1 January 2014 to 31 December 2016	−10.941	11.8481	0.7883	49.9635	7.0685	−0.124	−1.4036
	1 January 2014 to 30 June 2016 (Training)	−10.941	10.1515	0.1742	47.1743	6.8684	−0.0974	−1.4479
	1 July 2016 to 31 December 2016 (Testing)	−9.9938	11.8481	3.8317	52.6572	7.2565	−0.4786	−1.234
IMF7	1 January 2014 to 31 December 2016	−0.9518	1.2903	−0.0991	0.4445	0.6667	0.6826	−0.5425
	1 January 2014 to 30 June 2016 (Training)	−0.9518	1.2903	−0.0261	0.4801	0.6929	0.5038	−0.8375
	1 July 2016 to 31 December 2016 (Testing)	−0.8916	0.2486	−0.4609	0.1108	0.3328	0.5094	−0.959
IMF8	1 January 2014 to 31 December 2016	−0.1752	0.2321	0.0247	0.0217	0.1472	0.0304	−1.5499
	1 January 2014 to 30 June 2016 (Training)	−0.1749	0.2321	0.0593	0.0188	0.1371	−0.304	−1.3216
	1 July 2016 to 31 December 2016 (Testing)	−0.1752	−0.0809	−0.1463	0.0008	0.0281	0.7649	−0.6715
IMF9	1 January 2014 to 31 December 2016	−0.067	0.0673	0.0225	0.0016	0.0401	−0.6397	−0.8557
	1 January 2014 to 30 June 2016 (Training)	−0.067	0.0673	−0.0274	0.0005	0.0216	−0.138	−1.1845
	1 July 2016 to 31 December 2016 (Testing)	−0.067	0.0073	17.0341	0.3258	0.5708	−0.5572	−0.9412
Residue	1 January 2014 to 31 December 2016	15.7958	17.7251	17.0341	0.3258	0.5708	−0.5572	−0.9412
	1 January 2014 to 30 June 2016 (Training)	15.7958	17.6306	16.9026	0.2884	0.537	−0.4171	−1.0568
	1 July 2016 to 31 December 2016 (Testing)	17.6314	17.7251	17.6859	0.0008	0.0274	−0.337	−1.1062

^1^ SD, represents the standard deviation. The unit of minimum value, maximum value and mean value is °C.

## Appendix A

**Table A1:** Statistics of the original daily surface temperature data series and the decomposition results of Zhijiang station.

Series	Period	Min.	Max.	Mean	Variance	SD ^1^	Skewness	Kurtosis
Original data set	1 January 2014 to 31 December 2016	−0.7	31.8	18.0538	63.8016	7.9876	−0.2423	−1.1268
	1 January 2014 to 30 June 2016 (Training)	−0.7	30.85	17.5038	61.452	7.8391	−0.2228	−1.1137
	1 July 2016 to 31 December 2015 (Testing)	3.15	31.8	20.7796	66.5181	8.1559	−0.491	−1.1334
IMF1	1 January 2014 to 31 December 2016	−3.4876	3.3164	−0.0006	1.0313	1.0155	−0.006	0.5469
	1 January 2014 to 30 June 2016 (Training)	−3.4876	3.3164	−0.0038	1.0904	1.0442	0.0142	0.4111
	1 July 2016 to 31 December 2016 (Testing)	−2.6808	2.4776	0.0154	0.738	0.8591	−0.1602	1.4322
IMF2	1 January 2014 to 31 December 2016	−4.9666	4.6916	−0.0137	1.4461	1.2025	−0.0209	1.3251
	1 January 2014 to 30 June 2016 (Training)	−4.9666	4.6916	−0.0151	1.4498	1.2041	−0.0145	1.3825
	1 July 2016 to 31 December 2016 (Testing)	−3.5345	3.7363	−0.0067	1.4281	1.195	−0.0533	1.0959
IMF3	1 January 2014 to 31 December 2016	−3.9453	4.4972	−0.0368	1.5168	1.2316	0.0842	0.8224
	1 January 2014 to 30 June 2016 (Training)	−3.9453	4.4972	−0.0552	1.5108	1.2292	0.0978	0.899
	1 July 2016 to 31 December 2016 (Testing)	−3.6699	3.4617	0.0546	1.5364	1.2395	0.0151	0.5368
IMF4	1 January 2014 to 31 December 2016	−3.2607	3.8574	−0.0131	1.0952	1.0465	−0.0819	1.0059
	1 January 2014 to 30 June 2016 (Training)	−3.2607	3.8574	−0.0216	1.1767	1.0848	−0.0533	0.9823
	1 July 2016 to 31 December 2016 (Testing)	−2.0462	1.8588	0.029	0.6889	0.83	−0.2818	−0.2253
IMF5	1 January 2014 to 31 December 2016	−3.8762	5.271	0.0365	1.05	1.0247	0.3922	6.5503
	1 January 2014 to 30 June 2016 (Training)	−3.8762	5.271	0.0542	1.1226	1.0595	0.4193	6.897
	1 July 2016 to 31 December 2016 (Testing)	−1.2743	1.1222	−0.051	0.6811	0.8253	−0.1313	−1.4911
IMF6	1 January 2014 to 31 December 2016	−11.189	11.9534	0.7483	46.884	6.8472	−0.1075	−1.3765
	1 January 2014 to 30 June 2016 (Training)	−10.6116	10.1684	0.1262	42.3756	6.5097	−0.0985	−1.4462
	1 July 2016 to 31 December 2016 (Testing)	−11.189	11.9534	3.8314	57.8063	7.603	−0.5543	−1.1442
IMF7	1 January 2014 to 31 December 2016	−1.6727	1.9026	−0.0502	0.7532	0.8679	0.9044	−0.1937
	1 January 2014 to 30 June 2016 (Training)	−1.6727	1.9026	0.047	0.8221	0.9067	0.7038	−0.6052
	1 July 2016 to 31 December 2016 (Testing)	−0.9499	0.2884	−0.5321	0.1329	0.3645	0.6622	−0.7966
IMF8	1 January 2014 to 31 December 2016	−0.496	0.5448	0.0197	0.1395	0.3736	0.0206	−1.5351
	1 January 2014 to 30 June 2016 (Training)	−0.496	0.5448	0.0921	0.1335	0.3654	−0.3236	−1.362
	1 July 2016 to 31 December 2016 (Testing)	−0.4884	−0.0747	−0.339	0.0148	0.1216	0.569	−0.9077
IMF9	1 January 2014 to 31 December 2016	−0.0582	0.0586	0.0196	0.0012	0.0349	−0.6397	−0.8556
	1 January 2014 to 30 June 2016 (Training)	−0.0582	0.0586	0.0283	0.0009	0.0306	−1.1161	0.3131
	1 July 2016 to 31 December 2016 (Testing)	−0.0582	0.0068	−0.0235	0.0004	0.0189	−0.1403	−1.1839
Residue	1 January 2014 to 31 December 2016	16.2408	17.8054	17.345	0.2196	0.4686	−0.7969	−0.652
	1 January 2014 to 30 June 2016 (Training)	16.2408	17.8008	17.2537	0.2142	0.4628	−0.5833	−0.9155
	1 July 2016 to 31 December 2016 (Testing)	17.7763	17.8054	17.798	0.0001	0.0083	−1.1065	−0.0229

^1^ SD represents the standard deviation. The unit of minimum value, maximum value and mean value is °C.

## References

[1] Abdel-Aal R.E. (2004). Hourly temperature forecasting using abductive networks. Eng. Appl. Artif. Intell..

[2] Salcedo-Sanz S., Deo R.C., Carro-Calvo L., Saavedra-Moreno B. (2016). Monthly prediction of air temperature in Australia and New Zealand with machine learning algorithms. Theor. Appl. Climatol..

[3] Kim K.S., Taylor S.E., Gleason M.L., Koehler K.J. (2002). Model to enhance site-specific estimation of leaf wetness duration. Plant Dis..

[4] Yao Z., Lou G., Zeng X., Zhao Q. (2010). Research and Development Precision Irrigation Control System in Agricultural, Proceeding of the IEEE 2010 International Conference on Computer and Communication Technologies in Agriculture Engineering (CCTAE), Chengdu, China, 12–13 June 2010.

[5] Araghi A., Mousavi-Baygi M., Adamowski J., Martinez C., van der Ploeg M. (2017). Forecasting soil temperature based on surface air temperature using a wavelet artificial neural network. Meteorol. Appl..

[6] Kamarianakis Y., Ayuso S.V., Rodríguez E.C., Velasco M.T. (2016). Water temperature forecasting for Spanish rivers by means of nonlinear mixed models. J. Hydrol.: Reg. Stud..

[7] Karimi M., Vant-Hull B., Nazari R., Mittenzwei M., Khanbilvardi R. (2017). Predicting surface temperature variation in urban settings using real-time weather forecasts. Urban Clim..

[8] Ouellet-Proulx S., Chimi Chiadjeu O., Boucher M.-A., St-Hilaire A. (2017). Assimilation of water temperature and discharge data for ensemble water temperature forecasting. J. Hydrol..

[9] Benyahya L., Caissie D., St-Hilaire A., Ouarda T.B.M.J., Bobée B. (2007). A review of statistical water temperature models. Can. Water Resour. J..

[10] Piccolroaz S., Calamita E., Majone B., Gallice A., Siviglia A., Toffolon M. (2016). Prediction of river water temperature: A comparison between a new family of hybrid models and statistical approaches. Hydrol. Process..

[11] Sahoo G.B., Schladow S.G., Reuter J.E. (2009). Forecasting stream water temperature using regression analysis, artificial neural network, and chaotic non-linear dynamic models. J. Hydrol..

[12] Sohrabi M.M., Benjankar R., Tonina D., Wenger S.J., Isaak D.J. (2017). Estimation of daily stream water temperatures with a bayesian regression approach. Hydrol. Process..

[13] Toffolon M., Piccolroaz S. (2015). A hybrid model for river water temperature as a function of air temperature and discharge. Environ. Res. Lett..

[14] Attoue N., Shahrour I., Younes R. (2018). Smart building: Use of the artificial neural network approach for indoor temperature forecasting. Energies.

[15] Deihimi A., Orang O., Showkati H. (2013). Short-term electric load and temperature forecasting using wavelet echo state networks with neural reconstruction. Energy.

[16] Huddart B., Subramanian A., Zanna L., Palmer T. (2016). Seasonal and decadal forecasts of atlantic sea surface temperatures using a linear inverse model. Clim. Dyn..

[17] Khan M.Z.K., Sharma A., Mehrotra R. (2018). Using all data to improve seasonal sea surface temperature predictions: A combination-based model forecast with unequal observation lengths. Int. J. Climatol..

[18] Manzanas R., Gutiérrez J.M., Fernández J., van Meijgaard E., Calmanti S., Magariño M.E., Cofiño A.S., Herrera S. (2017). Dynamical and statistical downscaling of seasonal temperature forecasts in Europe: Added value for user applications. Clim. Serv..

[19] Yang Q., Wang M., Overland J.E., Wang W., Collow T.W. (2017). Impact of model physics on seasonal forecasts of surface air temperature in the Arctic. Mon. Weather Rev..

[20] Young P.C. (2018). Data-based mechanistic modelling and forecasting globally averaged surface temperature. Int. J. Forecast..

[21] Obrist D., Kirk J.L., Zhang L., Sunderland E.M., Jiskra M., Selin N.E. (2018). A review of global environmental mercury processes in response to human and natural perturbations: Changes of emissions, climate, and land use. Ambio.

[22] Slater L.J., Villarini G., Bradley A.A. (2017). Weighting of nmme temperature and precipitation forecasts across Europe. J. Hydrol..

[23] Zhao X.-H., Chen X. (2015). Auto regressive and ensemble empirical mode decomposition hybrid model for annual runoff forecasting. Water Resour. Manag..

[24] Deo R.C., Sahin M. (2016). An extreme learning machine model for the simulation of monthly mean streamflow water level in Eastern Queensland. Environ. Monit. Assess..

[25] Deo R.C., Şahin M. (2015). Application of the extreme learning machine algorithm for the prediction of monthly effective drought index in Eastern Australia. Atmos. Res..

[26] Deo R.C., Tiwari M.K., Adamowski J.F., Quilty J.M. (2017). Forecasting effective drought index using a wavelet extreme learning machine (W-elm) model. Stoch. Environ. Res. Risk A.

[27] Jiao G., Guo T., Ding Y. (2016). A new hybrid forecasting approach applied to hydrological data: A case study on precipitation in northwestern China. Water.

[28] Luo Y., Chang X., Peng S., Khan S., Wang W., Zheng Q., Cai X. (2014). Short-term forecasting of daily reference evapotranspiration using the hargreaves–samani model and temperature forecasts. Agric. Water Manag..

[29] Nastos P.T., Paliatsos A.G., Koukouletsos K.V., Larissi I.K., Moustris K.P. (2014). Artificial neural networks modeling for forecasting the maximum daily total precipitation at Athens, Greece. Atmos. Res..

[30] Wang W.C., Chau K.W., Qiu L., Chen Y.B. (2015). Improving forecasting accuracy of medium and long-term runoff using artificial neural network based on eemd decomposition. Environ. Res..

[31] Zhang X., Zhang Q., Zhang G., Nie Z., Gui Z. (2018). A hybrid model for annual runoff time series forecasting using elman neural network with ensemble empirical mode decomposition. Water.

[32] Balluff S., Bendfeld J., Krauter S. (2017). Meteorological data forecast using RNN. Int. J. Grid High Perf..

[33] Xu S., Niu R. (2018). Displacement prediction of baijiabao landslide based on empirical mode decomposition and long short-term memory neural network in three gorges area, China. Comput. Geosci..

[34] Yang Y.T., Dong J.Y., Sun X., Lima E., Mu Q.Q., Wang X.H. (2018). A CFCC-LSTM model for sea surface temperature prediction. IEEE Geosci. Remote Sens. Lett..

[35] Wei S., Yang H., Song J., Abbaspour K., Xu Z. (2013). A wavelet-neural network hybrid modelling approach for estimating and predicting river monthly flows. Hydrol. Sci. J..

[36] Chen B.F., Wang H.D., Chu C.C. (2007). Wavelet and artificial neural network analyses of tide forecasting and supplement of tides around Taiwan and South China sea. Ocean Eng..

[37] Nourani V., Alami M.T., Aminfar M.H. (2009). A combined neural-wavelet model for prediction of ligvanchai watershed precipitation. Eng. Appl. Artif. Intell..

[38] Pandey A.S., Singh D., Sinha S.K. (2010). Intelligent hybrid wavelet models for short-term load forecasting. IEEE Trans. Power Syst..

[39] Shafaei M., Kisi O. (2016). Lake level forecasting using wavelet-svr, wavelet-anfis and wavelet-arma conjunction models. Water Resour. Manag..

[40] Zhang F.P., Dai H.C., Tang D.S. (2014). A conjunction method of wavelet transform-particle swarm optimization-support vector machine for streamflow forecasting. J. Appl. Math..

[41] Torrence C., Compo G.P. (1997). A practical guide to wavelet analysis. Bull. Am. Meteorol. Soc..

[42] Wu Z., Huang N.E. (2009). Ensemble empirical mode decomposition: A noise-assisted data analysis method. Adv. Adapt. Data Anal..

[43] Huang N.E., Shen Z., Long S.R., Wu M.C., Shih H.H., Zheng Q., Yen N.C., Tung C.C., Liu H.H. (1998). The empirical mode decomposition and the hilbert spectrum for nonlinear and non-stationary time series analysis. Proc. R. Soc. A.

[44] Wang W.-C., Chau K.-W., Xu D.-M., Chen X.-Y. (2015). Improving forecasting accuracy of annual runoff time series using arima based on eemd decomposition. Water Resour. Manag..

[45] Zhang N., Lin A., Shang P. (2017). Multidimensionalk-nearest neighbor model based on eemd for financial time series forecasting. Physica A.

[46] Niu M., Gan K., Sun S., Li F. (2017). Application of decomposition-ensemble learning paradigm with phase space reconstruction for day-ahead PM_2.5_ concentration forecasting. J. Environ. Manag..

[47] Huang N.E., Shen Z., Long S.R. (1999). A new view of nonlinear water waves: The hilbert spectrum. Annu. Rev. Fluid Mech..

[48] Giles C.L., Lawrence S., Tsoi A.C. (2001). Noisy time series prediction using recurrent neural networks and grammatical inference. Mach. Learn..

[49] Kan M.S., Tan A.C.C., Mathew J. (2015). A review on prognostic techniques for non-stationary and non-linear rotating systems. Mech. Syst. Signal Proc..

[50] Rius A., Ruisánchez I., Callao M.P., Rius F.X. (1998). Reliability of analytical systems: Use of control charts, time series models and recurrent neural networks (RNN). Chemometr. Intell. Lab..

[51] Schmidhuber J. (2015). Deep learning in neural networks: An overview. Neural Netw..

[52] Bengio Y., Simard P., Frasconi P. (1994). Learning long-term dependencies with gradient descent is difficult. IEEE Trans. Neural Netw..

[53] Hochreiter S., Schmidhuber J. (1997). Long short-term memory. Neural Comput..

[54] Zhang X., Zhang Q., Zhang G., Gui Z. A comparison study of normalized difference water index and object-oriented classification method in river network extraction from landsat-tm imagery. Proceedings of the IEEE 2017 2nd International Conference on Frontiers of Sensors Technologies.

[55] Zhang X., Zhang Q., Zhang G., Nie Z., Gui Z. (2018). Landsat-based tow decades land cover change in Dongting Lake region. Fresen Environ. Bull..

[56] Kang A., Tan Q., Yuan X., Lei X., Yuan Y. (2017). Short-term wind speed prediction using EEMD-LSSVM model. Adv. Meteorol..

[57] Google Google Tensorflow. https://www.tensorflow.org/.

